# Multidimensional Discrimination Toward Single-Parent Families and Its Association With Depressive Symptoms of Parents: Cross-Sectional Study in South Korea

**DOI:** 10.2196/83771

**Published:** 2026-04-23

**Authors:** Seong-Uk Baek, Jin-Ha Yoon

**Affiliations:** 1 Graduate School, Yonsei University College of Medicine, Seodaemungu, Republic of Korea; 2 Department of Preventive Medicine, Yonsei University College of Medicine, 50-1 Yonsei-ro, Seodaemungu, 03722, Republic of Korea, 82 1034118697

**Keywords:** family, mental health, single father, single mother, single parenthood, stigma

## Abstract

**Background:**

Discrimination toward single-parent families (SPFs) is prevalent at structural and individual levels.

**Objective:**

This study examined the association between perceived discrimination toward SPFs and parental depressive symptoms in South Korea.

**Methods:**

This study included a nationally representative sample of 3300 single mothers (n=2205, 66.8%) and fathers (n=1095, 33.2%). Single parents’ perceptions of discrimination toward SPFs were measured using eight items evaluating the discrimination toward both participants and their children, which were categorized into four groups (lowest, low, high, and highest). Depressive symptoms were evaluated using the 9-item Patient Health Questionnaire. To examine the association between discrimination toward SPFs and depressive symptoms in single mothers and fathers, logistic regression models were used, and adjusted odds ratios and 95% CIs were calculated.

**Results:**

Of all participants, 11.7% (n=386) reported depressive symptoms. The prevalence of depressive symptoms was 7.7% (57/744), 6.4% (44/684), 8.2% (54/659), and 21.7% (155/714) among individuals with the lowest, low, high, and highest levels of discrimination, respectively. Compared to those experiencing the lowest level of discrimination, the highest level of discrimination was associated with 5.10-fold (95% CI 3.33‐7.79) and 6.12-fold (95% CI 2.80‐13.39) higher odds of depressive symptoms among single mothers and fathers, respectively. Further analyses demonstrated that discrimination directed toward both oneself and one’s children was associated with depressive symptoms.

**Conclusions:**

Discrimination against SPFs was prevalent in Korea and associated with depressive symptoms in both single mothers and fathers.

## Introduction

In recent decades, sociocultural shifts have increased societal interest in diverse family structures. These family forms, which often include civil partnerships, same-sex parent families, and single-parent families (SPFs), challenge the traditional and often narrowly defined concept of a family as consisting of a heterosexual couple and their biological children [1]. This shift reflects diverse ways in which individuals form meaningful familial relationships. In Korea, approximately 1.5 million households consisted of SPFs in 2023, accounting for 6.9% of total households [2]. In light of the growing interest in SPFs, the Korean government has implemented policies aimed at assisting child-rearing and providing financial resources for SPFs [3,4].

The mental health of SPF members has garnered substantial public health interest, as both single parents and their children are more vulnerable to various risk factors for mental health deterioration, including material deprivation, dual burden of work and childcare, and lack of social support [1,5,6]. For instance, previous studies have demonstrated that various factors, such as socioeconomic status, work-life balance, and social acceptance and support, can be associated with the mental health of single parents [5,7,8]. Indeed, single parents are more likely to have mood disorders and depressive symptoms than couple parents [9,10].

SPFs face various forms of discrimination at the institutional, structural, and individual levels [11]. In South Korea, influenced by Confucianism, which upholds the traditional family structure as the norm [12], cultural acceptance and institutional support for SPFs have progressed more slowly than in Western societies [13]. Parents of SPFs often experience stigmatization and discrimination in their daily lives, which negatively affects their mental well-being and overall health. For example, single mothers, particularly those who are divorced, face negative perceptions and stigmatization from neighbors and even close acquaintances [5,14]. Moreover, single parents are frequently stigmatized as having a lower socioeconomic status, rendering them more vulnerable to institutional discrimination in public and professional spheres than partnered parents [5,14,15]. Such stigmatization and discrimination can limit access to resources, employment opportunities, and social support systems [13].

Discrimination, irrespective of its nature, is a well-documented risk factor for poor mental health; however, the specific link between discrimination experienced by SPFs and depressive symptoms has been scarcely investigated. Experiencing discrimination can act as a major trigger for depressive symptoms by increasing chronic stress and lowering self-esteem [16]. Furthermore, social isolation and loneliness caused by discrimination can mediate the link between discrimination and depressive symptoms [17].

The existing literature on the mental health of single parents has some limitations. While studies have found that discrimination toward SPFs is linked to poor mental health among parents [7,18,19], most studies have primarily focused on single mothers, leaving important gaps in understanding sex-based differences in discrimination experiences and their association with depressive symptoms among single parents [20]. Therefore, based on a nationwide survey conducted in Korea, the objective of this study was to examine the association between perceived discrimination toward SPFs and the depressive symptoms of both single mothers and fathers by comprehensively assessing everyday discrimination experiences using a multidimensional discrimination measurement instrument. We hypothesized that perceived discrimination toward SPFs would be positively associated with depressive symptoms among single parents.

## Methods

### Study Population

The study sample was collected from the Single-Parent Family Survey, administered by the Korean Women’s Development Institute under the Ministry of Gender Equality and Family in Korea. The survey was conducted between August and November 2021. To ascertain a representative sample, the selection process involved probability proportional sampling of 331 administrative districts across South Korea, each containing at least eight single-parent households using the data from the 2019 Population and Housing Census. Systematic sampling was conducted within each district to select 8 to 15 households, and the household heads were surveyed. The survey response rate was 86.5%. If a selected household head refused to participate or remained unavailable despite repeated contact attempts, the household was classified as nonresponsive and replaced with an adjacent reserve household (13.5%). Consequently, 3300 parents of SPFs were included in the survey. In the survey, a single-parent household was defined as one in which the head of the household was divorced, widowed, never married, or separated, and the household included the head and at least one of their children aged 18 years or younger. A standardized survey weight was calculated for each individual to enhance the representativeness of the sample. Data collection involved structured one-on-one household interviews by trained interviewers conducted in compliance with COVID-19 prevention guidelines.

### Discrimination Toward SPFs

The perceived discrimination toward SPFs was evaluated in eight contexts. Specifically, participants were asked, “How often do you and your child experience unfair treatment or discrimination as a single-parent family? Please provide your responses from the perspectives of yourself and your child for each of the following contexts.” The first five items measured perceived discrimination toward participants in the following contexts: (1) neighborhood or community, (2) school or childcare facilities, (3) family and relatives, (4) workplace, and (5) public institutions. The remaining three items assessed participants’ perceived discrimination against their child in the following contexts: (1) neighborhood or community, (2) school or childcare facilities, and (3) family and relatives. To facilitate the participants’ understanding and ensure standardized responses, 2 to 3 examples were provided for each item, as detailed in Table S1 in Multimedia Appendix 1. Each item was rated on a 4-point Likert scale (1 “Never experienced discrimination,” 2 “Tended not to experience discrimination,” 3 “Tended to experience discrimination,” and 4 “Experienced severe discrimination”). Furthermore, participants could specify whether they had disclosed their single-parent status in each context rather than responding on a 4-point Likert scale. In such cases, the corresponding item was considered missing and replaced using multiple imputations. The total discrimination score ranged from 8 to 32, with higher scores indicating greater perceived discrimination toward SPFs. The Cronbach α was 0.93 for the sample. Based on the quartile values of the discrimination scores in the sample, participants were categorized into four groups: lowest (scores 8‐10), low (scores 11‐14), high (scores 15‐17), and highest (scores 18‐32) levels of discrimination.

### Depressive Symptoms

The Korean version of the 9-item Patient Health Questionnaire, a validated tool comprising nine items that evaluate mood and vitality over the past two weeks, was used to assess depressive symptoms [21,22]. Each item is scored on a scale ranging from 0 to 3, resulting in a total score between 0 and 27. Based on previous literature, a total score of 10 or more was classified as indicative of depressive symptoms [21,23].

### Covariates

The following sociodemographic features of the participants were considered confounders in the analyses: sex (male, female), age (<40 y, 40‐49 y, ≥50 y), education level (middle school or below, high school, college or above), marital status (divorced, others—separated, widowed, never married), monthly household income (<₩1,000,000, ₩1,000,000‐₩1,999,999, ₩2,000,000‐₩2,999,999, and ≥₩3,000,000; an exchange rate of ₩1000=US $0.67 is applicable), employment status (employed, unemployed), the number of children (one, two, three, or more), and physical activity. Physical activity was assessed using the following question: “How often do you currently engage in regular physical exercise (eg, walking, hiking, gym workouts, soccer, cycling, swimming)?” Participants who reported engaging in regular physical exercise 1 day or more per week were classified as physically active (yes), whereas all others were classified as no.

### Data Analysis

For the descriptive analysis, the sociodemographic characteristics of the study participants were examined according to their perceived discrimination levels. The prevalence of depressive symptoms was explored according to study variables. Moreover, the response frequency for each item was examined to analyze the distribution of discrimination responses for each context.

Multivariate logistic regression models were used to evaluate the association between discrimination of SPFs and depressive symptoms. Subsequently, discrimination was separated into two types: discrimination toward parents and discrimination toward children. Specifically, we assessed how an increase in the summed scores for the five items assessing discrimination toward parents and an increase in the three items assessing discrimination toward their children were associated with depressive symptoms. Adjusted odds ratios (ORs) and 95% CIs were determined. All analyses were not only conducted for the overall sample but also stratified by the sex of the parent to account for potential sex differences. Missing values were handled using multiple imputations, with 20 complete datasets generated through the multiple imputation by chained equations technique. Pooled estimates were calculated and presented. Statistical analyses and visualizations were conducted using R software (version 4.6.2; R Foundation for Statistical Computing). The complex survey design that involves systematic sampling was addressed using the “svydesign” and “svyglm” functions in the R package *survey* [24]. Subsequently, the results were combined using the *mice* package. We also assessed the robustness of our findings by performing a sensitivity analysis of all cases (n=2801). Finally, considering the wide range of scores in the highest category of perceived discrimination, it was further divided into 18 to 24 and 25 to 32 to examine whether a dose-response pattern could be observed.

### Ethical Considerations

This study was a secondary data analysis based on anonymized raw data from the Single-Parent Family Survey, which is publicly available at MicroData Integrated Service [25] without specific permission. To protect participants’ privacy and confidentiality, all data were anonymized and deidentified before the publication of the dataset by the Korean Women’s Development Institute. Therefore, the data provided to the authors were fully anonymized. Informed consent was provided by all participants during the original data collection procedure, and participants received no compensation for completing the survey. The Institutional Review Board (IRB) of the Yonsei Health System approved the study protocol (IRB application 2024-3489-002; IRB granting 4-2024-1607) and waived the requirement for additional informed consent because the study involved secondary analysis of existing data.

## Results

Table 1 shows the characteristics of the 3300 study participants. The sample consisted of 1095 (33.2%) male parents and 2205 (66.8%) female parents. Compared to those who experienced the lowest level of discrimination, individuals who reported higher levels of discrimination were more likely to be younger, have a marital status other than divorced, have lower income levels, be employed, and have only one child. However, no clear difference was noted in the proportions of male and female participants across the levels of perceived discrimination.

Table 2 presents the prevalence of depressive symptoms. Of the 3300 participants, 11.7% (n=386) had depressive symptoms. The prevalence of depressive symptoms was 7.7% (57/744), 6.4% (44/684), 8.2% (54/659), and 21.7% (155/714) among those with the lowest, low, high, and highest levels of discrimination, respectively. Furthermore, the prevalence of depressive symptoms was higher among women, those with low educational levels, those with low income levels, unemployed individuals, and individuals with three or more children.

**Table 1.** Sociodemographic features of the study participants. Single-Parent Family Survey, 2021, South Korea (cross-sectional study).

	Total (N=3300), n (%)	Perceived discrimination (n=2801)^a^, n (%)				*P* value
		Lowest (n=744)	Low (n=684)	High (n=659)	Highest (n=714)	
Sex						.60
Male	1095 (33.2)	256 (34.4)	224 (32.7)	236 (35.8)	236 (33.1)	
Female	2205 (66.8)	488 (65.6)	460 (67.3)	423 (64.2)	478 (66.9)	
Age (years)						.003
<40	722 (21.9)	153 (20.6)	139 (20.3)	155 (23.5)	167 (23.4)	
40‐49	2036 (61.7)	433 (58.2)	434 (63.5)	390 (59.2)	453 (63.4)	
≥50	542 (16.4)	158 (21.2)	111 (16.2)	114 (17.3)	94 (13.2)	
Education level						<.001
Middle school or below	120 (3.6)	27 (3.6)	19 (2.8)	36 (5.5)	20 (2.8)	
High school	1778 (53.9)	441 (59.3)	315 (46.1)	344 (52.2)	422 (59.1)	
College or above	1402 (42.5)	276 (37.1)	350 (51.2)	279 (42.3)	272 (38.1)	
Marital status						.047
Divorced	2684 (81.3)	591 (79.4)	551 (80.6)	535 (81.2)	606 (84.9)	
Others	616 (18.7)	153 (20.6)	133 (19.4)	124 (18.8)	108 (15.1)	
Income level (₩^b^)						<.001
<1,000,000	118 (3.6)	35 (4.7)	16 (2.3)	27 (4.1)	22 (3.1)	
1,000,000‐1,999,999	1030 (31.2)	274 (36.8)	168 (24.6)	179 (27.2)	226 (31.7)	
2,000,000‐2,999,999	1252 (37.9)	245 (32.9)	293 (42.8)	267 (40.5)	268 (37.5)	
≥3,000,000	900 (27.3)	190 (25.5)	207 (30.3)	186 (28.2)	198 (27.7)	
Employment status						.02
Employed	2680 (81.2)	585 (78.6)	577 (84.4)	549 (83.3)	596 (83.5)	
Unemployed	620 (18.8)	159 (21.4)	107 (15.6)	110 (16.7)	118 (16.5)	
Number of children						<.001
One	1842 (55.8)	367 (49.3)	375 (54.8)	380 (57.7)	417 (58.4)	
Two	1248 (37.8)	306 (41.1)	284 (41.5)	232 (35.2)	261 (36.6)	
Three or more	210 (6.4)	71 (9.5)	25 (3.7)	47 (7.1)	36 (5.0)	
Physical activity						.002
Yes	1472 (44.6)	322 (43.3)	293 (42.8)	323 (49.0)	277 (38.8)	
No	1828 (55.4)	422 (56.7)	391 (57.2)	336 (51.0)	437 (61.2)	

a Participants with missing values were excluded (n=499).

b An exchange rate of ₩1000=US $0.67 is applicable.

**Table 2.** Prevalence of depressive symptoms according to study variables. Single-Parent Family Survey, 2021, South Korea (cross-sectional study; N=3300).

	Depressive symptoms, n (%)		*P* value
	Yes	No	
Perceived discrimination			<.001
Lowest (n=744)	57 (7.7)	687 (92.3)	
Low (n=684)	44 (6.4)	640 (93.6)	
High (n=659)	54 (8.2)	605 (91.8)	
Highest (n=714)	155 (21.7)	559 (78.3)	
Missing values (n=499)	76 (15.2)	423 (84.8)	
Sex			.003
Male (n=1095)	102 (9.3)	993 (90.7)	
Female (n=2205)	284 (12.9)	1921 (87.1)	
Age (years)			.04
<40 (n=722)	66 (9.1)	656 (90.9)	
40‐49 (n=2036)	257 (12.6)	1779 (87.4)	
≥50 (n=542)	63 (11.6)	479 (88.4)	
Education level			<.001
Middle school or below (n=120)	19 (15.8)	101 (84.2)	
High school (n=1778)	245 (13.8)	1533 (86.2)	
College or above (n=1402)	122 (8.7)	1280 (91.3)	
Marital status			.80
Divorced (n=2684)	312 (11.6)	2372 (88.4)	
Others (n=616)	74 (12.0)	542 (88.0)	
Income level (₩)^a^			<.001
<1,000,000 (n=118)	33 (28.0)	85 (72.0)	
1,000,000‐1,999,999 (n=1030)	190 (18.4)	840 (81.6)	
2,000,000‐2,999,999 (n=1252)	122 (9.7)	1130 (90.3)	
≥3,000,000 (n=900)	41 (4.6)	859 (95.4)	
Employment status			<.001
Employed (n=2680)	218 (8.1)	2462 (91.9)	
Unemployed (n=620)	168 (27.1)	452 (72.9)	
Number of children			.03
One (n=1842)	220 (11.9)	1622 (88.1)	
Two (n=1248)	131 (10.5)	1117 (89.5)	
Three or more (n=210)	35 (16.7)	175 (83.3)	
Physical activity			<.001
Yes (n=1472)	229 (15.6)	1243 (84.4)	
No (n=1828)	157 (8.6)	1671 (91.4)	

a An exchange rate of ₩1000=US $0.67 is applicable.

Table 3 presents the response frequencies for the discrimination items. Approximately 13.4% to 18.3% of the participants reported tending to experience or experiencing severe discrimination. Specifically, 1.4% to 2.6% of the participants reported experiencing severe discrimination.

**Table 3.** Response frequencies on the discrimination questionnaire among the study participants. Single-Parent Family Survey, 2021, South Korea (cross-sectional study; N=3300).

	Never experienced discrimination, n (%)	Tended not to experience discrimination, n (%)	Tended to experience discrimination, n (%)	Experienced severe discrimination, n (%)	Did not disclose being a single parent, n (%)
Neighborhood or community	898 (27.2)	1450 (43.9)	476 (14.4)	75 (2.3)	401 (12.2)
Schools or childcare facilities	1135 (34.4)	1390 (42.1)	519 (15.7)	85 (2.6)	171 (5.2)
Family and relatives	1335 (40.5)	1304 (39.5)	457 (13.8)	78 (2.4)	126 (3.8)
Workplace	1244 (37.7)	1346 (40.8)	394 (11.9)	66 (2.0)	250 (7.6)
Public institutions	1240 (37.6)	1481 (44.9)	396 (12.0)	46 (1.4)	137 (4.1)
Neighborhood or community (child)	961 (29.1)	1355 (41.1)	545 (16.5)	51 (1.5)	388 (11.8)
Schools or childcare facilities (child)	1192 (36.1)	1385 (42.0)	505 (15.3)	47 (1.4)	171 (5.2)
Family and relatives (child)	1274 (38.6)	1444 (43.8)	417 (12.6)	41 (1.2)	124 (3.8)

Table 4 shows the association between perceived discrimination toward SPFs and parental depressive symptoms. Compared with those with the lowest discrimination level, those with the low (adjusted OR 1.64, 95% CI 1.05‐2.57), high (adjusted OR 1.74, 95% CI 1.12‐2.69), and highest (adjusted OR 5.33, 95% CI 3.65‐7.76) levels were more likely to have depressive symptoms. Furthermore, the highest discrimination level was associated with 6.12-fold (95% CI 2.80‐13.39) and 5.10-fold (95% CI 3.33‐7.79) increases in the odds of depressive symptoms in men and women, respectively.

**Table 4.** Association between discrimination toward single-parent families and depressive symptoms of parents. Single-Parent Family Survey, 2021, South Korea (cross-sectional study; n=3300). The models were adjusted for sex (overall model), age, education, marital status, income, employment status, number of children, and physical activity.

	Overall, adjusted OR^a^ (95% CI)	Male, adjusted OR (95% CI)	Female, adjusted OR (95% CI)
Perceived discrimination			
Lowest	Reference	Reference	Reference
Low	1.64 (1.05‐2.57)	1.94 (0.75‐5.00)	1.56 (0.94‐2.59)
High	1.74 (1.12‐2.69)	1.75 (0.75‐4.11)	1.79 (1.07‐2.97)
Highest	5.33 (3.65‐7.76)	6.12 (2.80‐13.39)	5.10 (3.33‐7.79)
Continuous scale			
8-point increase	3.26 (2.62‐4.05)	3.65 (2.27‐5.85)	3.14 (2.46‐4.01)

a OR: odds ratio.

**Figure 1.**
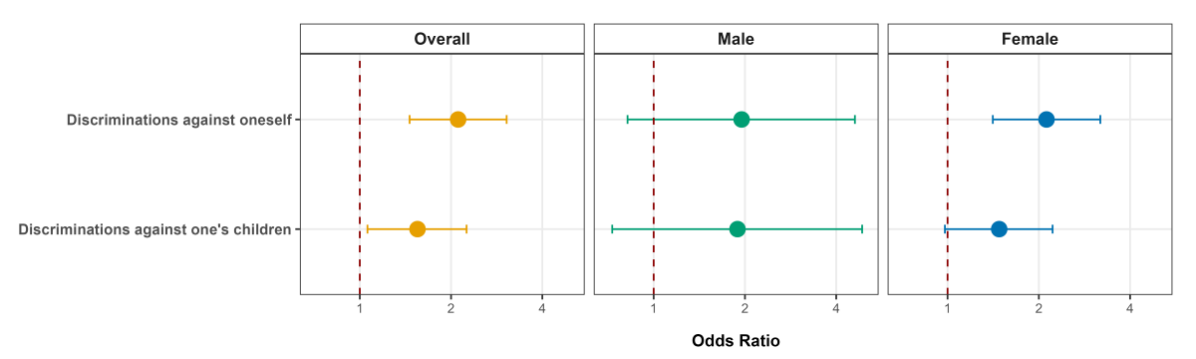
Associations between domains of perceived discrimination and depressive symptoms among single parents. The odds of depressive symptoms were estimated for a 5-point increase in discrimination toward oneself and a 3-point increase in discrimination toward one’s children. Single-Parent Family Survey, 2021, South Korea (cross-sectional study).

Figure 1 shows the associations between domains of perceived discrimination and depressive symptoms among single parents. A 5-point increase in discrimination toward oneself, as measured by the summed score, was associated with a 2.11-fold (95% CI 1.46‐3.05), 1.95-fold (95% CI 0.82‐4.61), and 2.12-fold (95% CI 1.41‐3.19) increase in the odds of depressive symptoms in the overall, male, and female samples, respectively. Similarly, a 3-point increase in discrimination toward one’s children, as measured by the summed score, was associated with a 1.55-fold (95% CI 1.06‐2.25), 1.89-fold (95% CI 0.73‐4.87), and 1.48-fold (95% CI 0.98‐2.22) increase in the odds of depressive symptoms in the overall, male, and female samples, respectively.

Table S2 in Multimedia Appendix 1 presents the association between discrimination toward SPFs and depressive symptoms in the complete cases. The findings of the sensitivity analysis were consistent with those of the main analysis, showing a similar positive association between discrimination experience and depressive symptoms. The results from the analyses further stratifying the highest category showed that, although the CIs were wide, notably elevated ORs were observed at the highest levels of discrimination, suggesting a dose-response pattern for this exposure category (Table S3 in Multimedia Appendix 1).

## Discussion

This study found an association between perceived discrimination toward SPFs and depressive symptoms in single parents. Single mothers and fathers who experienced the highest levels of discrimination were more likely to report depressive symptoms than those who experienced the lowest levels of discrimination. Furthermore, discrimination directed towards both parents and their children was positively associated with parents’ depressive symptoms.

Descriptive analysis revealed that 11.7% (386/3300) of the participants presented with depressive symptoms. This figure is notably higher than the prevalence of depressive symptoms measured by the 9-item Patient Health Questionnaire in nationally representative Korean samples, which ranged from 2.8% to 5.2% [26,27]. Furthermore, while a substantial number of single parents reported experiencing discrimination across various domains, clear sex differences in their levels of discrimination were not evident.

Our findings align with those of the existing literature, showing an association between experiences of discrimination and mental health problems such as depressive symptoms, suicidal behaviors, and anxiety [28,29]. Furthermore, the results of our study are consistent with those of previous research, showing that discrimination toward minority family structures is associated with poor mental health outcomes in affected individuals. For instance, discrimination targeting families with same-sex marriage has been linked to adverse mental health outcomes for themselves and their children [30,31]. Moreover, perceived discrimination negatively correlated with happiness among unmarried mothers [19]. Although various forms of discrimination, including those based on sex and race/ethnicity, have consistently been shown to be associated with mental health problems [28,29], this study adds to the literature by exploring the relationship between discrimination directed toward SPFs and depressive symptoms.

Complex mechanisms may explain the association between perceived discrimination toward SPFs and depressive symptoms of single parents. Multiple pathways may link the experience of discrimination to mental health deterioration, and the existing literature suggests that discrimination can induce psychological, physiological, and behavioral responses that contribute to poor health outcomes [16]. Prolonged exposure to discrimination can lead to chronic psychological distress by inducing anxiety regarding exclusion or by reducing self-esteem [32,33]. The persistence of stress can eventually result in the development of depressive symptoms. Moreover, physiologically, chronic stress arising from the experience of discrimination was linked to the dysregulation of the hypothalamic-pituitary-adrenal axis, which manifests as increased cortisol levels [34,35]. Hypothalamic-pituitary-adrenal axis overactivity plays a key role in the pathophysiology of depressive symptoms [36]. Finally, the discrimination experienced by SPFs may lead to behavioral changes. For instance, experiencing chronic stress may induce individuals to rely on coping mechanisms, such as smoking or alcohol consumption, which can contribute to the deterioration of mental health [37].

This study had certain limitations. First, the causal effects of discrimination toward SPFs on depressive symptoms could not be identified, as the analyses were based on cross-sectional survey data. For instance, the possibility of reverse causality, in which depressive symptoms influence the perception of discrimination [38], could not be ruled out in this cross-sectional study. Therefore, longitudinal studies are needed to examine whether experiences of discrimination can predict the onset of depressive symptoms over time. Second, key variables, including depressive symptoms, were assessed using self-reported measures, which may be subject to measurement errors, such as recall bias. Future studies should consider using medical records or physician diagnoses to verify the association between discrimination and objective clinical diagnoses. Third, certain factors that could influence both discrimination and depressive symptoms, such as psychiatric history, personality, social support, neighborhood income levels, alcohol use, smoking status, and other health behaviors, were not accounted for due to the lack of available information. We acknowledge that the failure to account for these potential unmeasured confounders may have resulted in an overestimation of the association between discrimination experiences and depressive symptoms. Fourth, our discrimination survey questionnaire aimed to capture experiences of discrimination toward SPFs across various daily contexts. However, as it relies on subjective recall, it may be subject to recall bias. Additionally, the survey responses demonstrated high reliability, the instrument has not undergone formal validation. Because the measure was not externally validated, it may be conceptually limited and may not comprehensively capture discrimination toward SPFs. Additionally, the subjective nature of the measure may introduce systematic bias if individuals with depressive symptoms differentially report higher levels of perceived discrimination in comparison to those without [38]. Therefore, future research should establish a theoretical framework for understanding discrimination experiences among SPFs and develop a validated measurement tool to overcome these limitations. Fifth, although this study addressed missing data using multiple imputation, the assumption of missing at random could not be verified. We acknowledge that missingness may have been influenced by unobserved socioeconomic or health-related factors; therefore, the results derived from multiple imputation may still be subject to bias. Sixth, although this study aimed to explore sex-specific associations between discrimination toward SPFs and depressive symptoms, the relatively small sample size of single fathers resulted in wider CIs and less stable estimates compared with those observed among single mothers. Future epidemiological studies may address this limitation by including a larger sample of single fathers.

Despite these limitations, this study contributes to existing literature in several ways. First, the study sample was selected through systematic sampling, representing a nationally representative population, which enhances the generalizability of the findings. Second, our study provides novel insights by analyzing the scarcely studied association between discrimination toward SPFs and mental health.

This study showed that single parents experience discrimination toward both themselves and their children owing to their single-parent status, which is closely associated with depressive symptoms. An association between perceived discrimination toward SPFs and depressive symptoms was noted in both single mothers and fathers.

## Supplementary material

10.2196/83771 Multimedia Appendix 1 Survey questionnaire on the perceived discrimination toward single-parent families, sensitivity analyses based on the complete sample, and the results of the analysis further stratifying the highest category.

10.2196/83771 Checklist 1 STROBE checklist.

## Abbreviations

IRB: Institutional Review Board
OR: odds ratio
SPF: single-parent family

## References

[1] Jensen TM, Sanner C (2021). A scoping review of research on well‐being across diverse family structures: rethinking approaches for understanding contemporary families. J Family Theo Revie.

[2] (2023). The lives of men and women in 2023: a statistical overview [Web Page in Korean]. Ministry of Gender Equality and Family.

[3] Jang MS, Lee YH (2016). Policy needs & improvements for single-parent families childcare [Article in Korean]. Korean Fam Resour Manag Assoc.

[4] Chin M, Lee J, Lee S, Son S, Sung M (2012). Family policy in South Korea: development, current status, and challenges. J Child Fam Stud.

[5] Vo T, Canty L (2023). Global mental health experiences of single mothers: a mixed methods research synthesis. J Adv Nurs.

[6] Strickland JR (2024). The relationship of food insecurity and mental health in single-parent households: a literature review. URNCST J.

[7] Kareem OM, Oduoye MO, Bhattacharjee P (2024). Single parenthood and depression: a thorough review of current understanding. Health Sci Rep.

[8] Dharani MK, Balamurugan J (2024). The psychosocial impact on single mothers’ well-being - a literature review. J Educ Health Promot.

[9] Kim KJ, Yoo JH (2019). Single mothers and mental health in South Korea: the Seventh Korea National Health and Nutrition Examination Survey, 2016. Korean J Fam Pract.

[10] Subramaniam M, Prasad RO, Abdin E, Vaingankar JA, Chong SA (2014). Single mothers have a higher risk of mood disorders. Ann Acad Med Singap.

[11] Choi S, Byoun SJ, Kim EH (2020). Unwed single mothers in South Korea: Increased vulnerabilities during the COVID-19 pandemic. Int Soc Work.

[12] Park IH, Cho LJ (1995). Confucianism and the Korean Family. J Comp Fam Stud.

[13] Park H, Choi J, Jo H (2016). Living arrangements of single parents and their children in South Korea. Marriage Fam Rev.

[14] Rusyda HM, Lukman ZM, Subhi N (2011). Coping with difficulties: social inequality and stigmatization on single mothers with low income household. Pertanika J Soc Sci Humanit.

[15] Lauster N, Easterbrook A (2011). No room for new families? A field experiment measuring rental discrimination against same-sex couples and single parents. Soc Probl.

[16] Williams DR, Lawrence JA, Davis BA, Vu C (2019). Understanding how discrimination can affect health. Health Serv Res.

[17] Han S, Hu Y, Wang L (2021). Perceived discrimination and mental health symptoms among persons living with HIV in China: the mediating role of social isolation and loneliness. AIDS Care.

[18] Kim A, Jeon S, Song J (2023). Self-stigma and mental health in divorced single-parent women: mediating effect of self-esteem. Behav Sci (Basel).

[19] Thasleema A, Rajan SK (2022). Perceived discrimination and happiness among tribal unmarried mothers. Psychol Stud.

[20] Kim JH (2024). The effect of discrimination on depression in single-parent household heads [Article in Korean]. J Fam Resour Manag Policy Rev.

[21] Kroenke K, Spitzer RL, Williams JB (2001). The PHQ-9: validity of a brief depression severity measure. J Gen Intern Med.

[22] Park SJ, Choi HR, Choi JH, Kim KW, Hong JP (2010). Reliability and validity of the Korean version of the Patient Health Questionnaire-9 (PHQ-9) [Article in Korean]. Anxiety Mood.

[23] Levis B, Benedetti A, Thombs BD, DEPRESsion Screening Data (DEPRESSD) Collaboration (2019). Accuracy of Patient Health Questionnaire-9 (PHQ-9) for screening to detect major depression: individual participant data meta-analysis. BMJ.

[24] Lumley T (2004). Analysis of complex survey samples. J Stat Soft.

[25] MicroData Integrated Service.

[26] Kim SY, Yoo DM, Min C, Choi HG (2022). Assessment of the difference in depressive symptoms of the Korean adult population before and during the COVID-19 pandemic using a community health survey. J Affect Disord.

[27] Lee EJ, Kim SJ (2023). Prevalence and related factors of depression before and during the COVID-19 pandemic: findings from the Korea National Health and Nutrition Examination Survey. J Korean Med Sci.

[28] Vines AI, Ward JB, Cordoba E, Black KZ (2017). Perceived racial/ethnic discrimination and mental health: a review and future directions for social epidemiology. Curr Epidemiol Rep.

[29] Vargas SM, Huey SJ, Miranda J (2020). A critical review of current evidence on multiple types of discrimination and mental health. Am J Orthopsychiatry.

[30] Donnelly R, Robinson BA, Umberson D (2019). Can spouses buffer the impact of discrimination on depressive symptoms? An examination of same-sex and different-sex marriages. Soc Ment Health.

[31] Mazrekaj D, Jin Y (2023). Mental health of children with gender and sexual minority parents: a review and future directions. Humanit Soc Sci Commun.

[32] Urzúa A, Ferrer R, Godoy N (2018). The mediating effect of self-esteem on the relationship between perceived discrimination and psychological well-being in immigrants. PLoS ONE.

[33] Brandt L, Liu S, Heim C, Heinz A (2022). The effects of social isolation stress and discrimination on mental health. Transl Psychiatry.

[34] Lee DB, Peckins MK, Miller AL (2021). Pathways from racial discrimination to cortisol/DHEA imbalance: protective role of religious involvement. Ethn Health.

[35] Mikulska J, Juszczyk G, Gawrońska-Grzywacz M, Herbet M (2021). HPA axis in the pathomechanism of depression and schizophrenia: new therapeutic strategies based on its participation. Brain Sci.

[36] Vreeburg SA, Hoogendijk WJG, van Pelt J (2009). Major depressive disorder and hypothalamic-pituitary-adrenal axis activity: results from a large cohort study. Arch Gen Psychiatry.

[37] Rosario M, Schrimshaw EW, Hunter J (2011). Cigarette smoking as a coping strategy: negative implications for subsequent psychological distress among lesbian, gay, and bisexual youths. J Pediatr Psychol.

[38] Liu H, Yang TC (2022). Examining the reciprocity between perceived discrimination and health: a longitudinal perspective. Popul Res Policy Rev.

