# Supplementary material for: Multidimensional Discrimination Toward Single-Parent Families and Its Association With Depressive Symptoms of Parents: Cross-Sectional Study in South Korea
Source: JMIR Public Health Surveill. 2026 Apr 23;12:e83771. doi: 10.2196/83771 (PMC13105235; doi:10.2196/83771)
Supplement: Multimedia Appendix 1 [file publichealth-v12-e83771-s001.docx]

**Table S1.** Survey questionnaire on the perceived discrimination toward single-parent families

| **Items** |  |
| --- | --- |
| **Question** | How often do you and your child experience unfair treatment or discrimination as a single-parent family? Please provide your responses from the perspectives of both yourself and your child for each of the following contexts: |
| **1.** | **Neighborhood or community**  Examples:   - Being denied rental or lease agreements owing to being a single parent - Losing contact with others after disclosing single-parent status - Facing negative perceptions or prejudices toward single parents |
| **2.** | **Schools or childcare facilities**  Examples:   - Experiencing disadvantages in access or services owing to single-parent status - Being excluded from parent meetings or events - Encountering events that assume two-parent families as the norm |
| **3.** | **Family and relatives**  Examples:   - Being excluded from family decision-making processes owing to single-parent status - Hearing insulting or derogatory remarks about single parents |
| **4.** | **Workplace**  Examples:   - Facing hiring discrimination or denial of leave or vacation owing to single-parent status - Experiencing ostracism from colleagues |
| **5.** | **Public institutions**  Examples:   - Experiencing cold treatment owing to single-parent status - Having personal privacy exposed in public spaces |
| **6.** | **Neighborhood or community (regarding your child)**  Examples:   - Your child losing contact with peers after disclosing their single-parent family status - Negative attitudes or prejudices toward your child owing to single-parent status |
| **7.** | **Schools or childcare facilities (regarding your child)**  Examples:   - Being excluded from peer groups or events - Your child experiencing disadvantages in access or services owing to single-parent status - Encountering events that assume two-parent families as the norm |
| **8.** | **Family and relatives (regarding your child)**  Examples:   - Your child being excluded from family decision-making processes - Hearing insulting or derogatory remarks directed at your child for being from a single-parent family |

**Table S2** Sensitivity analyses based on the complete cases (N = 2,801)

|  | **Overall** | **Male** | **Female** |
| --- | --- | --- | --- |
|  | **OR (95% CI)** | **OR (95% CI)** | **OR (95% CI)** |
| **Perceived discrimination** |  |  |  |
| Lowest | Reference | Reference | Reference |
| Low | 1.29 (0.79–2.09) | 1.98 (0.75–5.27) | 1.10 (0.63–1.91) |
| High | 1.59 (0.99–2.54) | 1.89 (0.79–4.53) | 1.56 (0.90–2.72) |
| Highest | 4.98 (3.38–7.33) | 5.88 (2.63–13.16) | 4.76 (3.07–7.39) |
| **Continuous scale** |  |  |  |
| 8-point increase | 3.24 (2.57–4.08) | 3.46 (2.12–5.64) | 3.16 (2.44–4.09) |

OR, odds ratio; CI, confidence interval
The models were adjusted for sex (overall model), age, education, marital status, income, employment status, the number of children, and physical activity

**Table S3** The results of the analysis further stratifying the highest category

|  | **Overall** | **Male** | **Female** |
| --- | --- | --- | --- |
|  | **OR (95% CI)** | **OR (95% CI)** | **OR (95% CI)** |
| **Perceived discrimination** |  |  |  |
| Lowest | Reference | Reference | Reference |
| Low | 1.65 (1.05–2.58) | 1.94 (0.75–5.02) | 1.57 (0.95–2.60) |
| High | 1.73 (1.12–2.69) | 1.75 (0.74–4.13) | 1.78 (1.07–2.97) |
| Highest – 1 | 4.19 (2.83–6.19) | 4.73 (2.06–10.84) | 4.03 (2.60–6.26) |
| Highest – 2 | 20.67 (11.43–37.39) | 31.06 (9.61–100.36) | 17.82 (9.10–34.89) |

OR, odds ratio; CI, confidence interval
The models were adjusted for sex (overall model), age, education, marital status, income, employment status, the number of children, and physical activity
